# Finite element analysis of posterior acetabular column plate and posterior acetabular wall prostheses in treating posterior acetabular fractures

**DOI:** 10.1186/s13018-023-03535-9

**Published:** 2023-02-11

**Authors:** Guixiong Huang, Yizhou Wan, Kaifang Chen, Zhenchun Yin, Qinghua Song, Yi Xu, Xiaodong Guo

**Affiliations:** 1 Department of Orthopaedics, Union Hospital, Tongji Medical College, Huazhong University of Science and Technology, Wuhan, Hubei People’s Republic of China; 2 Department of Plastic and Reconstructive Surgery, Ningbo First Hospital, Ningbo, People’s Republic of China

**Keywords:** Acetabular posterior wall fracture, Acetabular posterior column plate, Acetabular posterior wall prosthesis, Finite element analysis

## Abstract

**Background:**

The purpose of this study was to investigate the mechanical stability of the posterior acetabular column plate and different posterior acetabular wall prostheses used in treating posterior acetabular fractures with or without comminution.

**Methods:**

The unilateral normal ilium was reconstructed, and a model of posterior acetabular wall fracture was established on this basis. The fracture fragment accounted for approximately 40% of the posterior acetabular wall. The posterior acetabular column plate and different posterior acetabular wall prostheses were also designed. Using static and dynamic analysis methods, we observed and compared the changes in the stress and displacement values of different models at different hip joint flexion angles under external forces.

**Results:**

At different hip flexion angles, the stress of each model mainly fluctuated between 37.98 MPa and 1129.00 MPa, and the displacement mainly fluctuated between 0.076 and 6.955 mm. In the dynamic analysis, the nodal stress‒time curves of the models were nonlinear, and the stress changed sharply during the action time. Most of the nodal displacement‒time curves of the models were relatively smooth, with no dramatic changes in displacement during the action time; additionally, most of the curves were relatively consistent in shape.

**Conclusions:**

For simple posterior acetabular wall fractures, we recommend using a posterior acetabular column plate. In the case of comminuted posterior acetabular fractures, we recommend the use of a nonflanked posterior acetabular prosthesis or a biflanked posterior acetabular prosthesis. Regarding the method of acetabular prosthesis design, we propose the concept of “Break up to Make up” as a guide.

## Background

Posterior acetabular fractures are the most common type of acetabular fracture, accounting for approximately 15–47% of acetabular fractures [1–4], and are commonly caused by car accidents or falls from heights [5]. Currently, reconstructing the integrity of the posterior wall of the acetabulum is difficult. Many complications may occur after posterior acetabular wall fracture, with the most common complications including traumatic hip arthritis, femoral head necrosis, hip instability and redislocation [6, 7], which can greatly increase the financial burden on patients. At present, for common posterior acetabular wall fractures, if the fracture is a simple posterior wall fracture without fragmentation, the original fragment is used for reduction. If the fracture fragment is comminuted, then autologous ilium reconstruction is applied; however, because this method cannot achieve complete reconstruction of the acetabular articular surface, the probability of postoperative hip osteoarthritis or femoral head necrosis is relatively high.

To solve the problem of reconstructing the posterior wall of the acetabulum, we designed a plate for the posterior column of the acetabulum, which is mainly intended for use in treating simple posterior wall fractures of the acetabulum. To solve the problems related to comminuted fractures of the posterior acetabular wall, we designed three types of posterior acetabular prostheses, with the aim of reconstructing the articular surface of the posterior acetabular wall and maintaining the anatomical reduction of the posterior acetabular wall to the greatest extent possible. The purpose of this study was to understand and compare the mechanical response of the above different methods for the internal fixation of posterior acetabular fractures and to select the best internal fixator according to the type of posterior acetabular fracture. We use the finite element method to compare the forces in different models at different hip flexion angles using static and dynamic analysis to understand the mechanical properties of the different models.

## Methods

**Fig. 1:**
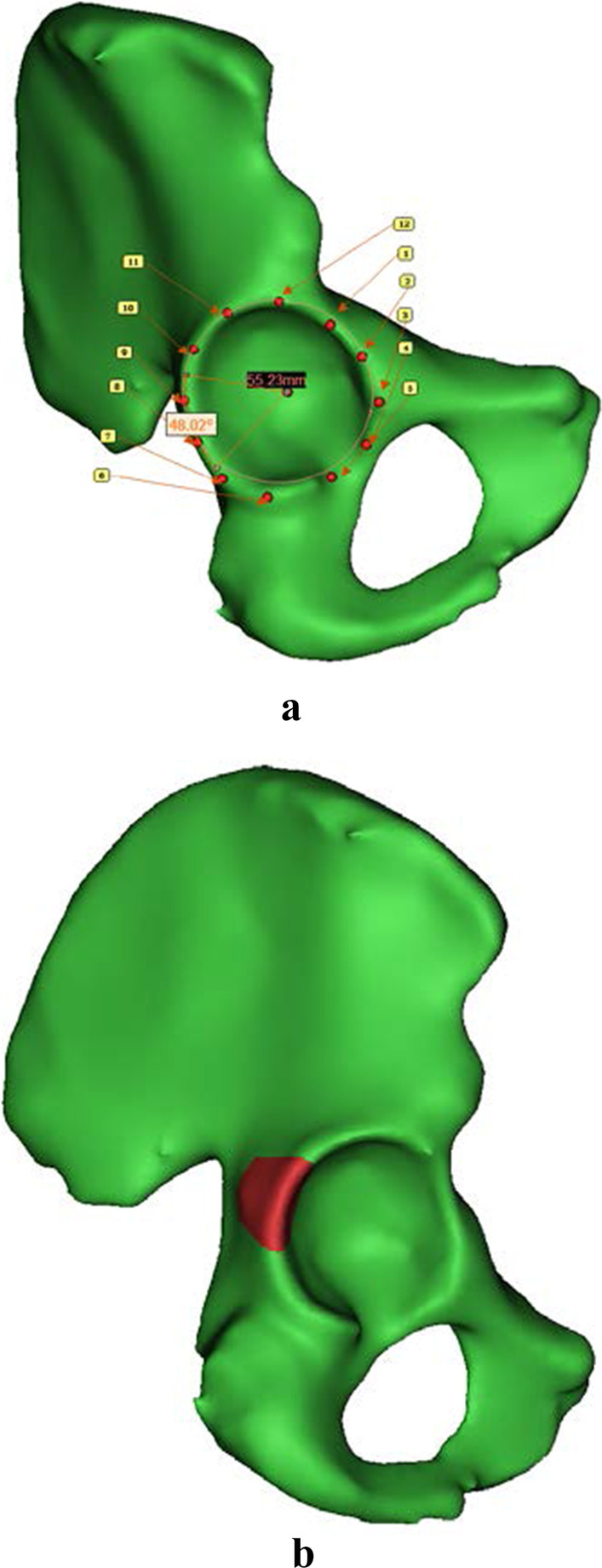
**a** Method of posterior acetabular wall division and schematic diagram showing that the fracture fragment accounts for 40% of the posterior acetabular wall. **b** Model of the posterior acetabular wall

**Fig. 2:**
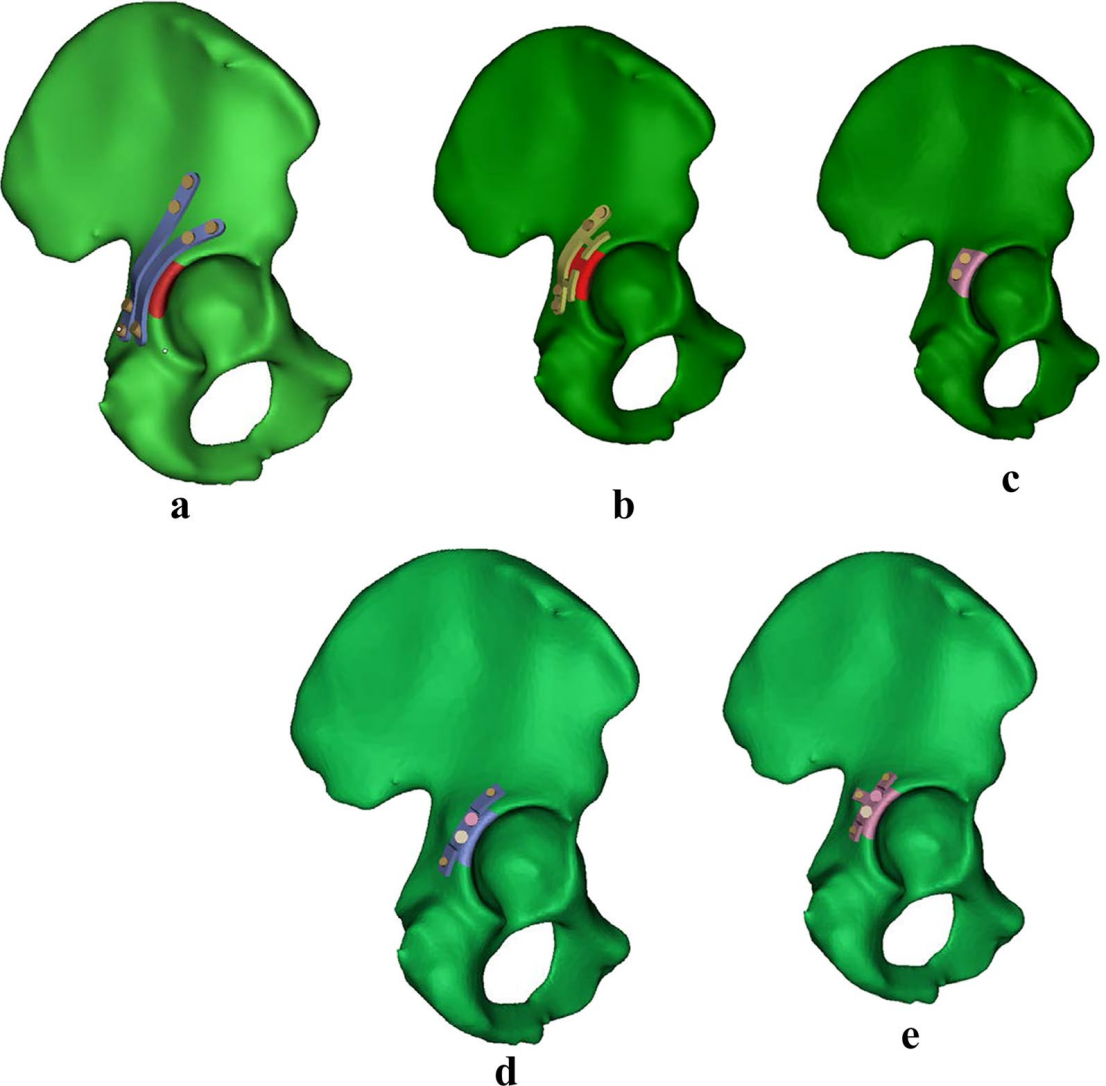
**a** CRP model. **b** PAPC model. **c** NPAWP model. **d** BPAWP model. **e** TPAWP model. CPR: common reconstruction plate, PACP: posterior acetabular column plate, NPAWP: nonflanked posterior acetabular wall prosthesis, BPAWP: biflanked posterior acetabular wall prosthesis, TPAWP: triflanked posterior acetabular wall prosthesis

Computed tomography (CT) data of a volunteer’s hip were obtained and imported into Mimics 20.0 software (Materialise, Leuven, Belgium) to reconstruct a normal ilium. The acetabular fossa of the ilium was divided into 12 equal parts (clock model) and marked as 12 points, with the 12th point corresponding to the intersection of the line connecting the anteroinferior iliac spine and the ischial tuberosity with the top of the acetabulum, and the sixth point symmetrical to the 12th point symmetrical across the centre of the acetabulum. Each point was named according to the corresponding point on a clock. The posterior wall ranged from the 7th point to the 11th point, accounting for 120°; in this ilium model, the diameter of the acetabular fossa was 55.23 mm. Then, a fragment was created in the posterior wall of the acetabulum, accounting for approximately 40% of the posterior wall, i.e., 48°, as shown in Fig. 1. After the posterior acetabular wall fracture model was established, with or without comminution, different models for fixing the posterior wall of the acetabulum were established, including the following prosthesis models: a common reconstruction plate (CRP) model, consisting of two medial and lateral reconstruction plates for posterior wall fracture fixation; a posterior acetabular column plate (PACP) model, consisting of a main plate and two small upper and lower “T” plates on the same side; a nonflanked posterior acetabular wall prosthesis (NPAWP) model; a biflanked posterior acetabular wall prosthesis (BPAWP) model, consisting of two (upper and lower) lateral wings; and a triflanked posterior acetabular wall prosthesis (TPAWP) model, consisting of three (upper, middle and lower) lateral wings. The model of the ilium was imported into 3-matic to construct the models of the steel plates and prostheses. To reduce the complexity of the analysis, the model was simplified by ignoring the soft tissue around the hip joint and the femoral head. The final result is shown in Fig. 2. The above five models were then imported into HyperMesh 13.0 software (Altair Company, Troy, MI, USA) for meshing using four-node tetrahedral elements (C3D4). Then, the separate fracture model was imported into Mimics 20.0 software for material assignment. We applied titanium alloy material properties for the steel plates and prostheses and smoothly polished the hip joint surface of the prostheses. Finally, all models were imported into Abaqus 2021 software (Dassault; France) for analysis. Static and dynamic analyses were carried out to obtain numerical values for stress and displacement. In the dynamic analysis, plots of stress versus time and displacement versus time were also obtained.

### Material properties

The plates, screws and prostheses in this study were all assigned the properties of titanium alloy, with a Young modulus of 110 GPa and Poisson ratio of 0.316 [8]. It was assumed that the materials of the internal fixators, prostheses and ilium were all isotropic. When assigning material properties to the iliac bone model, we followed our previous method [8], based on the CT value. According to the formula, different CT values correspond to different Young’s moduli; thus, the true material properties of the ilium can be approximated. In the static analysis, surface‒surface contact, hard contact tangent behaviour, a penalty contact attribute and a friction coefficient between bone and bone of 0.40 were applied [8]; surface‒surface contact, hard contact tangent behaviour, a penalty contact property and a friction coefficient between the internal fixators or prostheses and the ilium of 0.45 were applied [8]. As boundary conditions, the pubic symphysis, articular surface of the ilium and sacroiliac joint were fixed. In the dynamic analysis, general contact (display dynamic analysis), hard contact tangent behaviour and a penalty contact attribute were applied. The mechanical analysis allowed only one type of contact to exist; thus, only the friction between the internal fixators or prostheses and the ilium was set to 0.45, and the friction between bone and bone was ignored.

### Boundaries and loads

Loading was applied in two ways. In the static analysis, the five models were simulated in the standing position and at 30°, 60°, 90° and 120° of hip joint flexion and were subjected to a force of 2300 N [9]; the stress distribution of the internal fixators and the displacement of the models were determined under all these conditions. In the dynamic analysis, at 90° of hip flexion, the femoral head was simulated to impact the posterior wall of the acetabulum with the same force. The force increased from 0 to 2300 N in the first 0.5 ms and decreased to 0 in the last 0.5 ms. The total duration of force application was 1 ms [10], representing the mechanical response of contact with the posterior wall of the acetabulum. The relationships between the maximum stress and displacement nodes and times for the five model plates and screws or prostheses and screws were obtained.

### Statistical analysis

To compare whether the stress and displacement of different models were significantly different, the Kruskal‒Wallis test method was used in this study, and *P* < 0.05 was considered statistically significant. If a significant difference between the models was calculated, pairwise comparisons were performed using a multivariate ANOVA method.

## Results

A CRP model (592,718 units, 133,777 nodes), an APCP (450,007 units, 100,879 nodes), an NPAWP model (466,968 units, 101,596 nodes), a BPAWP model (440,890 units, 97,965 nodes) and a TPAWP model (488,085 units, 108,440 nodes) were established.

In the static and dynamic analyses, the CRP, APCP, NPAWP, BPAWP and TPAWP models were placed in different positions, and the maximum stress in each position is shown in Table 1. The maximum stress of each plate and prosthesis model fluctuated between 37.98 and 1129.00 MPa. The maximum displacement is shown in Table 2. In different positions and under different conditions, the displacement of the models fluctuated between 0.076 mm and 6.955 mm. The static displacement results are similar to those of the in vitro biomechanical study reported by Altun et al. [9], supporting the credibility of the experimental results of this study.

**Table 1:** Maximum stress of five models in different positions (MPa)

Models and body positions	CRP model	PAPC model	NPAWP model	BPAWP model	TPAWP model
Standing (statics)	198.27	59.73	46.86	92.94	192.65
30° of hip flexion (statics)	83.99	78.07	256.41	246.61	177.25
60° of hip flexion (statics)	120.97	88.26	109.32	142.38	98.06
90° of hip flexion (statics)	149.57	137.37	168.44	340.97	210.36
120° of hip flexion (statics)	146.05	155.81	325.28	448.59	308.15
Standing (dynamics)	255.72	206.30	395.25	328.44	689.73
30° of hip flexion (dynamics)	135.77	103.27	317.31	1028.63	1192.00
60° of hip flexion (dynamics)	93.76	37.98	181.04	119.12	872.97
90° of hip flexion (dynamics)	126.60	444.55	692.86	188.53	766.17
120° of hip flexion (dynamics)	183.50	708.74	1050.35	255.47	1034.23

**Table 2:** Maximum displacement of five models in different positions (mm)

Models and body positions	CRP model	PAPC model	NPAWP model	BPAWP model	TPAWP model
Standing (statics)	0.233	0.194	0.263	0.184	0.240
30° of hip flexion (statics)	0.104	0.103	0.092	0.076	0.083
60° of hip flexion (statics)	0.336	0.318	0.292	0.269	0.296
90° of hip flexion (statics)	0.578	0.517	0.526	0.468	0.512
120° of hip flexion (statics)	0.656	0.580	0.621	0.542	0.593
Standing (dynamics)	0.241	0.380	0.205	0.198	0.232
30° of hip flexion (dynamics)	0.077	0.092	0.272	0.068	0.075
60° of hip flexion (dynamics)	0.456	0.343	0.900	0.246	0.296
90° of hip flexion (dynamics)	1.381	2.001	4.283	0.999	1.137
120° of hip flexion (dynamics)	1.832	3.139	6.955	1.618	1.757

**Fig. 3:**
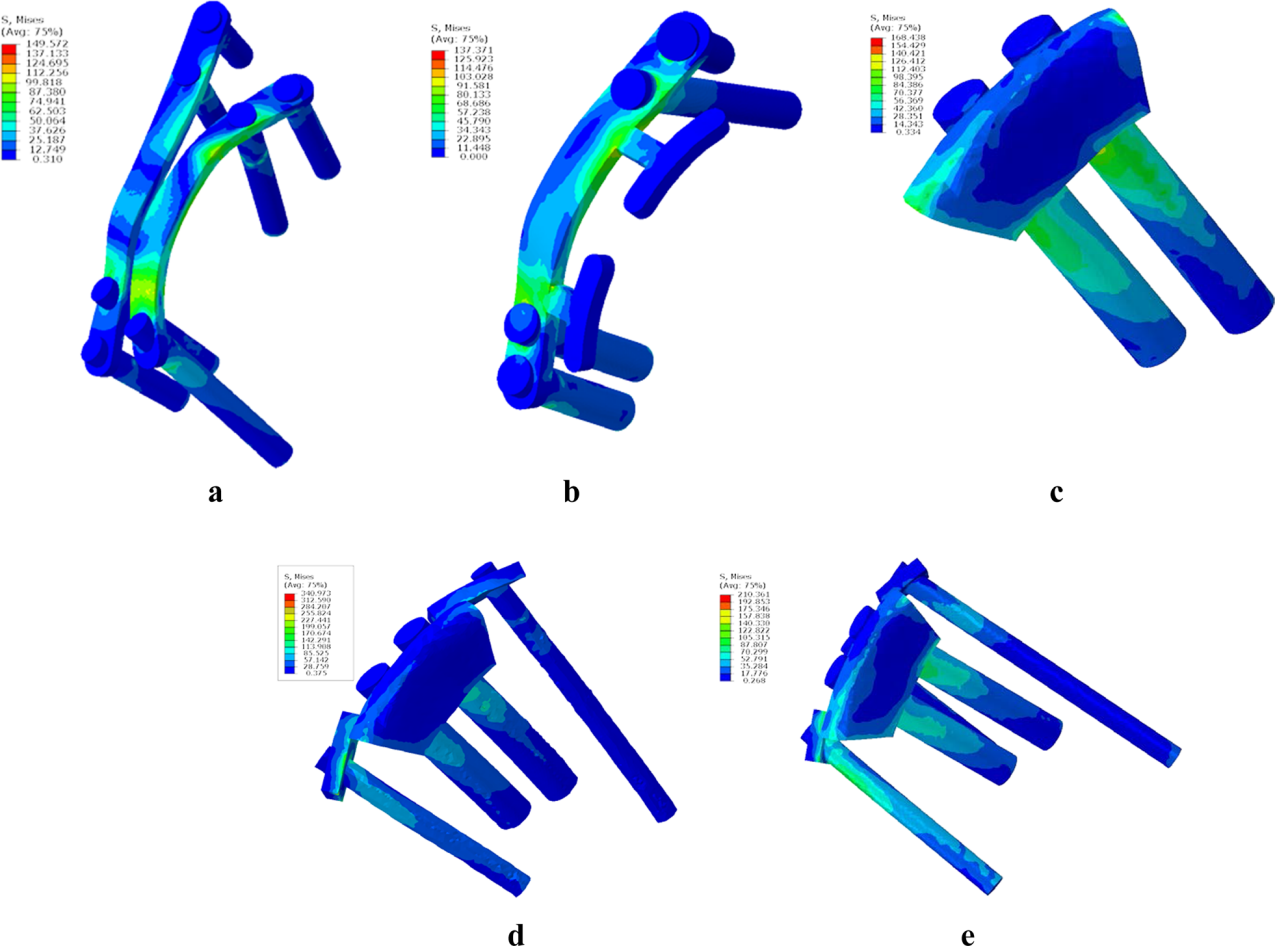
Stress distribution of each model at 90° of hip flexion in the static analysis. **a** CRP model. **b** PACP model. **c** NPAWP model. **d** BPAWP model. **e** TPAWP model. CPR: common reconstruction plate, PACP: posterior acetabular column plate, NPAWP: nonflanked posterior acetabular wall prosthesis, BPAWP: biflanked posterior acetabular wall prosthesis, TPAWP: triflanked posterior acetabular wall prosthesis

**Fig. 4:**
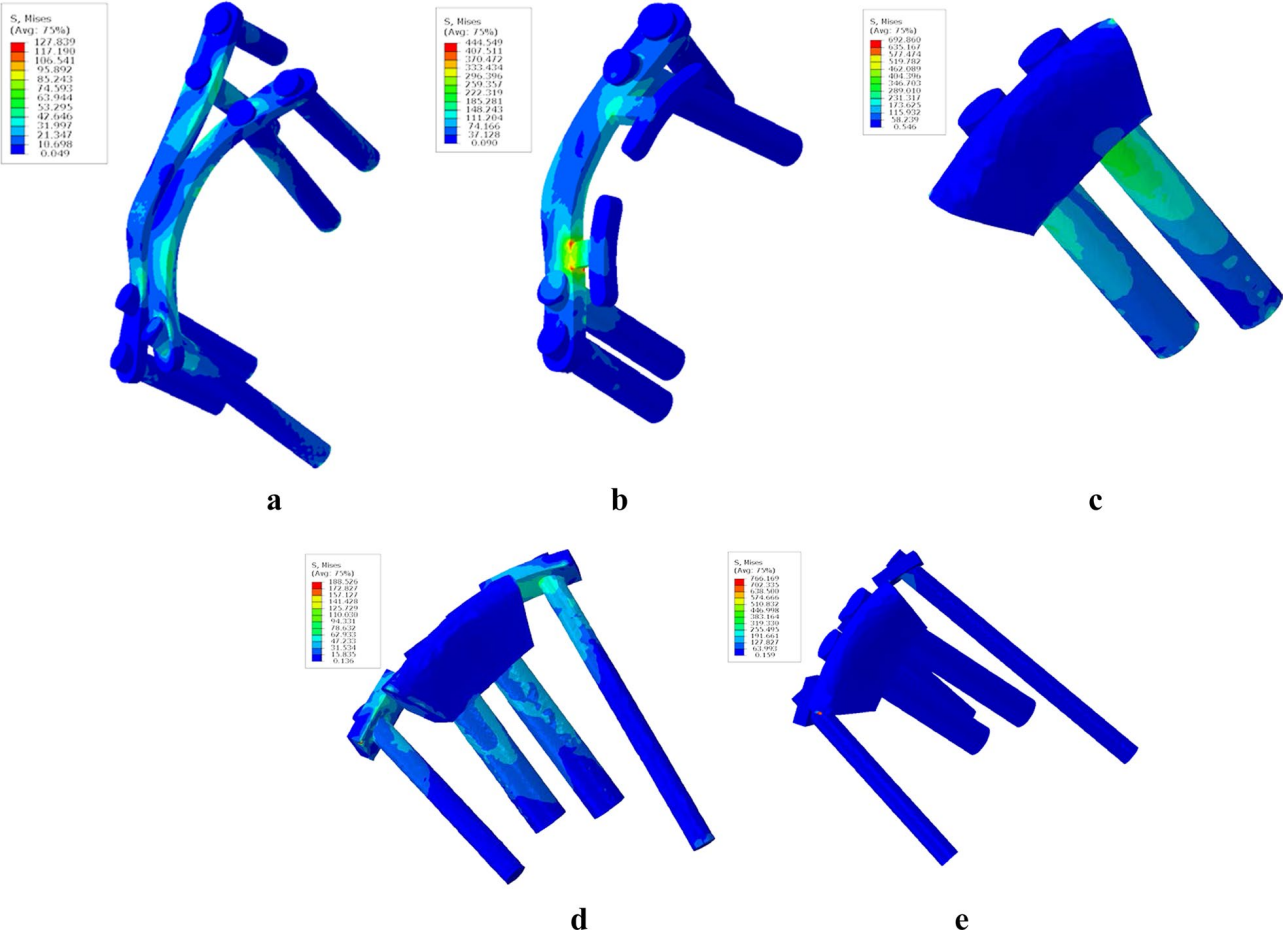
Stress distribution of each plate–screw or prosthesis–screw model at 90° of hip flexion in the dynamic analysis. **a** CRP model. **b** PACP model. **c** NPAWP model. **d** BPAWP model. **e** TPAWP model. CPR: common reconstruction plate, PACP: posterior acetabular column plate, NPAWP: nonflanked posterior acetabular wall prosthesis, BPAWP: biflanked posterior acetabular wall prosthesis, TPAWP: triflanked posterior acetabular wall prosthesis

The stress distributions of different models in the static and dynamic analyses are shown in Figs. 3 and 4, respectively. In this study, the stress distribution was affected by the degree of hip flexion as well as the force. As the hip flexed, the distribution of the maximum stress was mostly vertical, along the internal fixator or prosthesis around the acetabular joint. Additionally, stress was commonly found at the junction of the plate and screw hole, plate and bone surface, “T” plate and main plate, prosthesis and screw and prosthesis and bone surface.

**Fig. 5:**
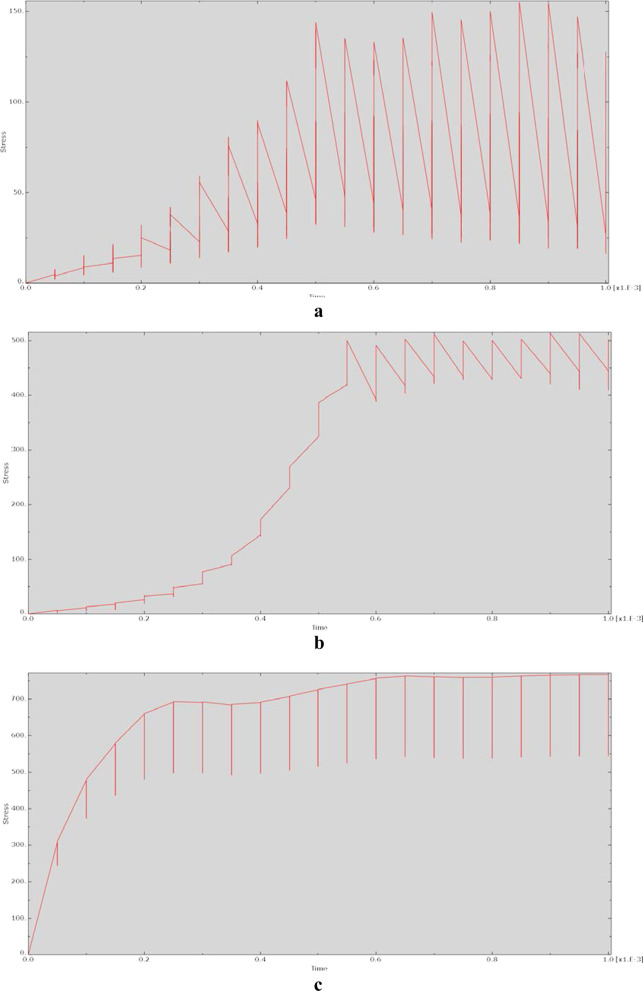

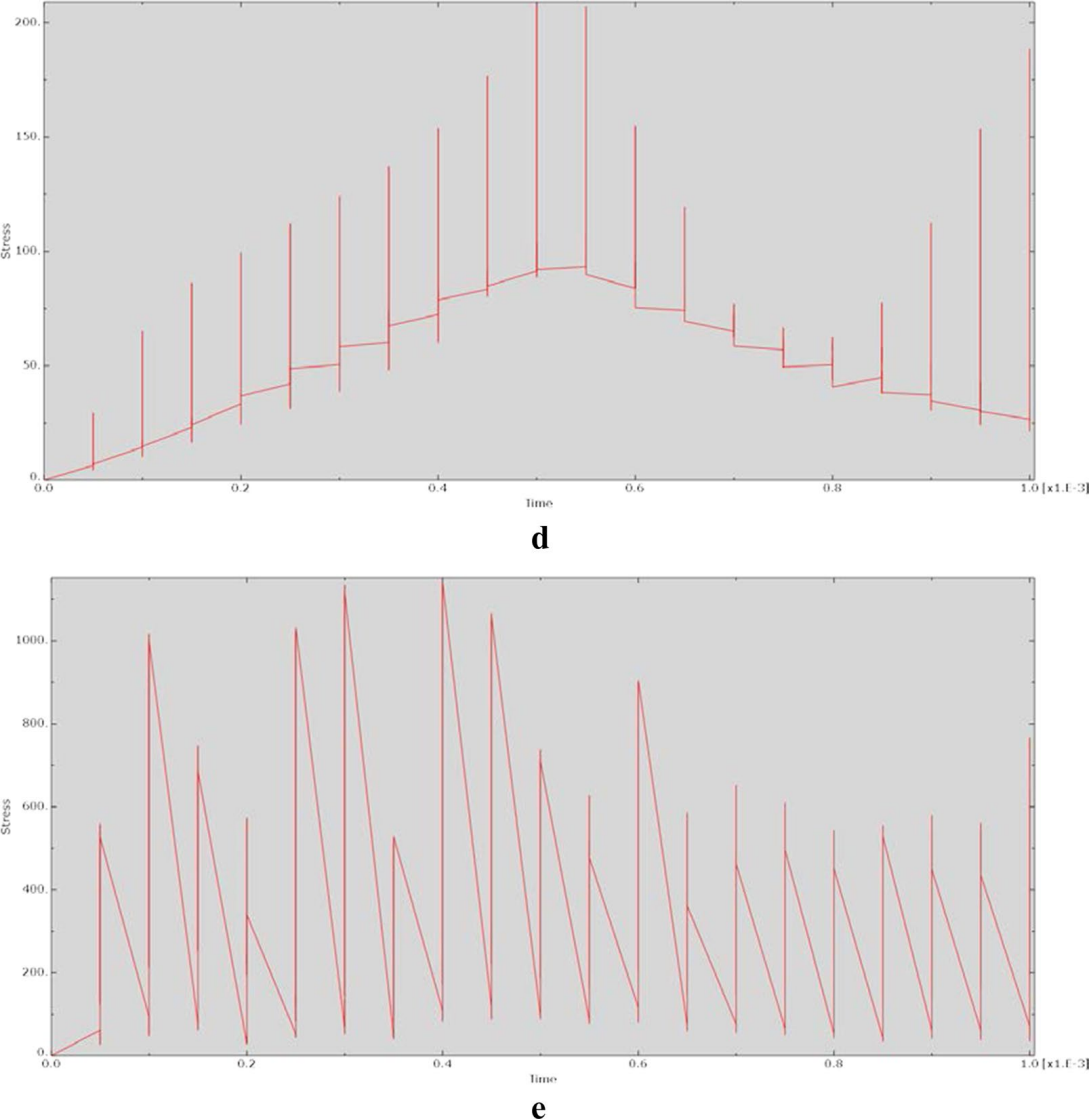
Relationship between stress and time for each model at 90° of hip flexion in the dynamic analysis. **a** CRP model. **b** PACP model. **c** NPAWP model. **d** BPAWP model. **e** TPAWP model. CPR: common reconstruction plate, PACP: posterior acetabular column plate, NPAWP: nonflanked posterior acetabular wall prosthesis, BPAWP: biflanked posterior acetabular wall prosthesis, TPAWP: triflanked posterior acetabular wall prosthesis

The relationship between stress and action time during the dynamic analysis is shown in Fig. 5. The relationship between stress and time was irregular, with the relationship for each model showing a unique and irregular shape. However, many of the model stress‒time plots exhibited a sawtooth-like shape.

**Fig. 6:**
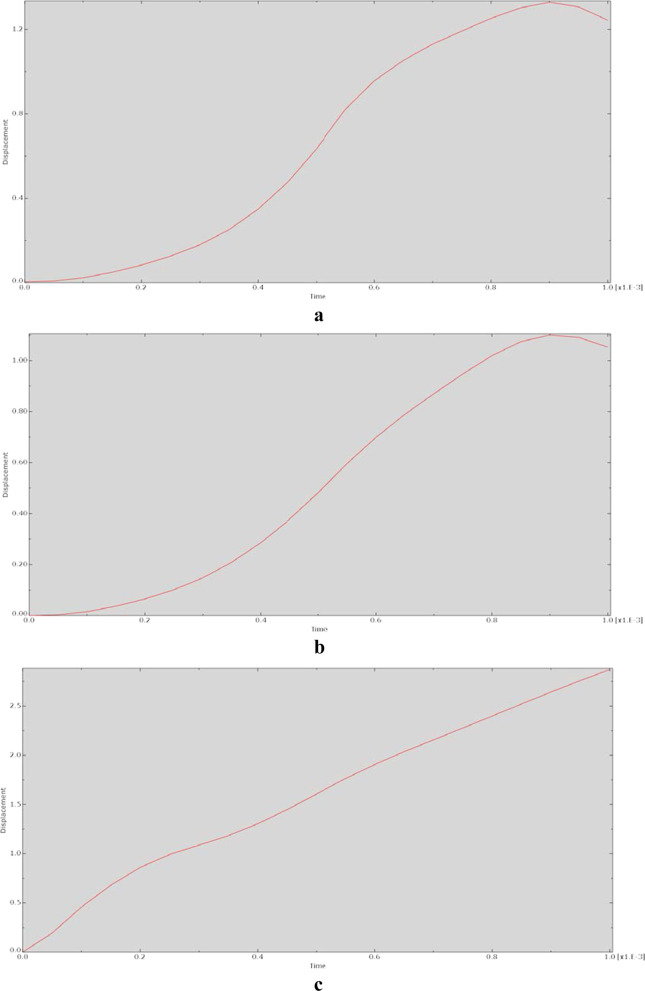

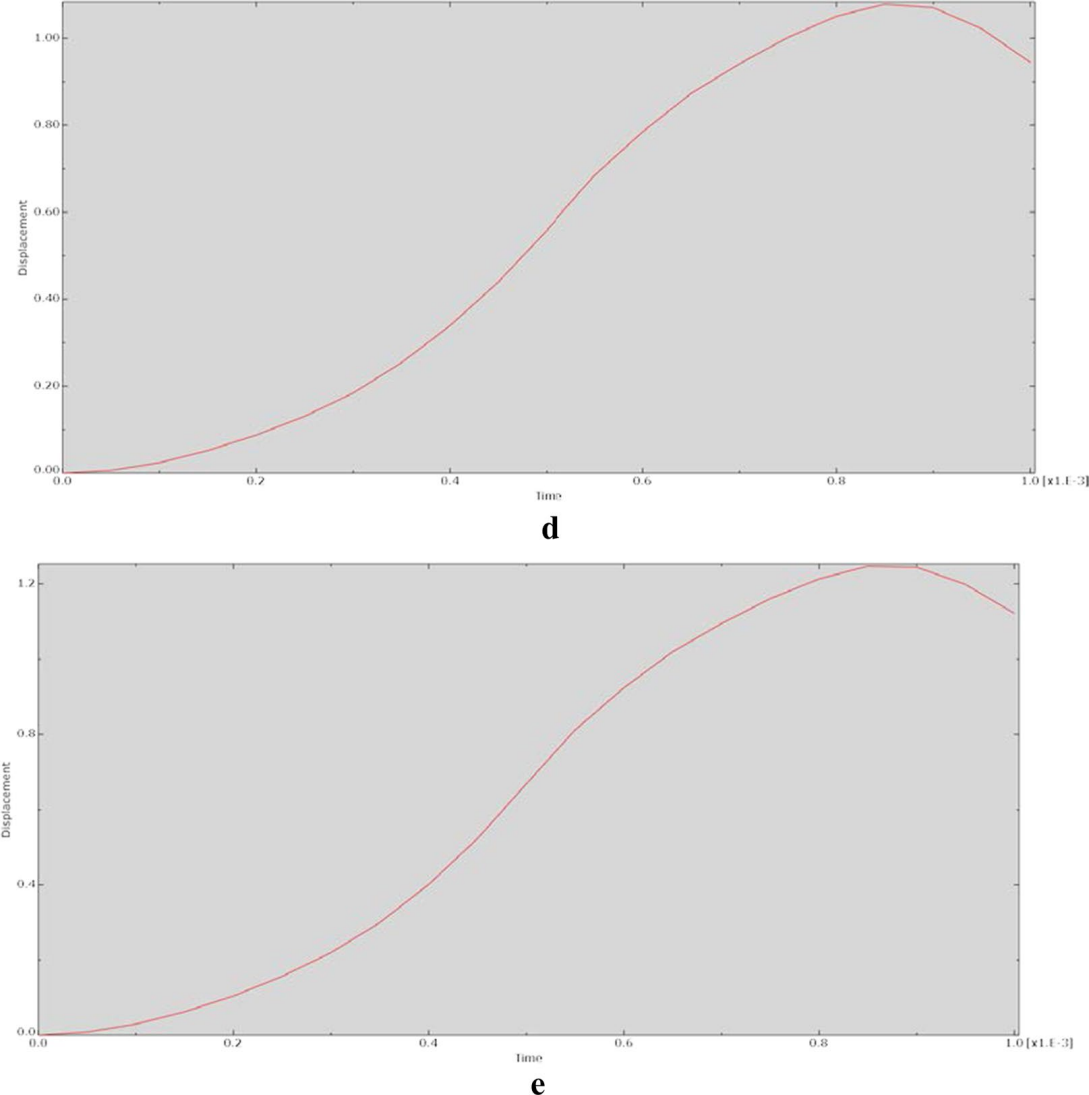
Relationship between displacement and time for each model at 90° of hip flexion in the dynamic analysis. **a** CRP model. **b** PACP model. **c** NPAWP model. **d** BPAWP model. **e** TPAWP model. CPR: common reconstruction plate, PACP: posterior acetabular column plate, NPAWP: nonflanked posterior acetabular wall prosthesis, BPAWP: biflanked posterior acetabular wall prosthesis, TPAWP: triflanked posterior acetabular wall prosthesis

The relationship between displacement and action time during the dynamic analysis is shown in Fig. 6. The graphs of the displacement–time relationship for each model were essentially the same, indicating nonlinear relationships.

Statistical analysis using the Kruskal‒Wallis test showed a significant difference in stress among the models (*P* = 0.005 < 0.05) but not displacement (*P* = 0.792 > 0.05). When the displacement data were subjected to multivariate analysis of variance, the pairwise comparisons between different models indicated a statistically significant difference between the CRP model and the TPAWP model (*P* = 0.02 < 0.05). There were no significant differences between the other models.

## Discussion

The results of this study suggest that the stress of the plate and prosthesis models ranges between 37.98 and 1129.00 MPa. According to the statistical analysis results, the magnitude of the stress varied significantly among the models, mainly between the CRP and TPAWP models. There were other differences between the models. The stress values of the TPAWP model in various positions were larger than those of the other models, indicating that the design of that prosthesis was more likely to cause stress concentration. If this prosthesis was subjected to the complex mechanical environment in vivo, the design may lead to an increased risk of internal fixation failure. In this study, titanium material properties were applied, and the yield strength of titanium is between 800 and 1000 MPa [11]. Applied forces exceeding this range will likely cause internal fixation fracture. According to our research results, when the hip joint was flexed at 30°, 60° and 120°, the stress of the acetabular prosthesis models fell within this range or exceeded this range, indicating that when the external force is 2300 N, the acting time of the force is 1 ms, and the hip joint is flexed at one of the above angles, loosening of or damage to the prosthesis may easily occur. We recommend avoiding strenuous movements with the hip in flexion when using this type of prosthesis. The research of the static and dynamic analyses of the hip joint showed that under different degrees of hip flexion, the displacement fluctuated from 0.076 to 6.953 mm, and statistical analysis revealed no significant differences. This finding indicates that the models in this study preliminarily meet the requirements of internal fixation stability under the applied load type.

The maximum stress of the models was mainly observed at the edge of the plate, at the edge of the screw holes, at the junction between the “T”-shaped components and the main plate and at the junction between the flank and the bone surface. The common features of areas of stress concentration were sudden changes in the geometric shape and cuts in the material of the components [12]. On this basis, repeated loading during activities of daily life may cause fatigue damage [13]. Therefore, to improve the design of our plates and prostheses, some additional processing can be performed in places where the shape suddenly changes, such as the application of some circular chamfering structures to serve as a transitional structure between different shapes and mitigate stress concentration.

Most of the stress‒time curves exhibited a sawtooth-like structure, indicating that after the node was acted on by an external force, the stress of the node changed violently and nonlinearly. The reason for this may be that the dynamic behaviour of the same material is unstable and varies with the loading parameters [14]. Moreover, the output of this study is represented in terms of changes at the nodes of the models, and these changes may be more obvious at the node level than at the model component level. In the displacement‒time relationship diagrams, the changes at the nodes were relatively smooth. Except for the curve of the prosthesis model (e.g., the “CRP model”) being close to linear, the curves of other models were not very different and were relatively smooth, showing an “S” shape.

The stress and displacement results indicate that the stress of the prostheses is relatively concentrated relative to the stress of the plate and that the stress of the TPAWP model is larger than that of the other models, with a significant difference compared with the CRP model. We preliminarily conclude that for internal fixation stability and surgical success, other internal fixation methods should be given priority. For example, if there is a noncomminuted fracture of the posterior acetabular wall, the PACP should be preferred because the plate is anatomically designed to avoid the need to prebend the plate during the operation, which would require additional operation time and increase the risk of the operation. In addition, the PACP features two “T”-shaped plates to reinforce the posterior wall fracture fragments, and each “T”-shaped plate has screw holes (the actual product has 3 screw holes; the model was simplified in this study) and can be fixed with screws as needed. Both the NPAWP and the BPAWP can be used to reconstruct the anatomy of the posterior acetabular wall. However, the maximum stress demonstrated by the NPAWP in this study was 1050.35 MPa, while the maximum stress demonstrated by the BPAWP was 1028.63 MPa, indicating that the BPAWP may have better mechanical stability than NPAWP. Structurally, the NPAWP has a simple design and allows a good mechanical effect to be achieved with only two screws. Under the premise of avoiding strenuous exercise after surgery, to save operation time, the NPAWP is recommended.

In actual clinical practice, to create a posterior acetabular prosthesis that fits the shape of the fractured posterior wall of the acetabulum to the greatest extent, we recommend individualizing the design of the prosthesis and printing it; additionally, an osteotomy guide plate should be designed at the same time to appropriately remove a part of the bone such that the prosthesis fits the posterior wall of the acetabulum to the greatest extent. Furthermore, the prosthesis should be designed with a porous structure [15], and bone tissue engineering strategies can be applied to create a scaffold [16]. The purpose of these strategies is to maximize the preservation of bone mass and facilitate future hip replacement. Under the premise of strength, the pore size needs to be maximized for autologous bone grafting to retain sufficient bone mass. At the same time, to improve the design of the prosthesis, absorbable metals can be used [17] in the fabrication process. However, it is necessary to ensure that absorbable material remains in the body for a sufficiently long period. When reconstructing the acetabular articular surface, to prevent the prosthesis from rubbing against the femoral head, the articular surface of the prosthesis needs to be polished and the edges chamfered. Alternatively, on the premise of ensuring the strength of the prosthesis, the articular surface of the prosthesis can be designed with a thin layer of titanium and a baffle at the rear of the prosthesis to support the prosthesis and prevent the femoral head from affecting the prosthesis under physiological conditions. Such a baffle structure can dissipate dangerous kinetic energy [18]. The posterior part of the prosthesis can consist of a frame structure, which is filled in with grafted bone. In this case, several baffle-like structures are needed, and screws can be passed through the flank structures to fix the prosthesis. The main advantages of these prosthesis design concepts are as follows: anatomically reconstructing the posterior wall of the acetabulum; maintaining the stability of the acetabulum; maintaining the bone volume of the posterior wall via the frame structure; and avoiding wear of the femoral head by polishing the articular surface. Li et al. [19] claimed that reconstructing an acetabular with large defects was a challenge during surgery. In some complex cases, the key to success was how to attach the fixations to the remaining autologous bone. The advantage of individualized acetabular components was that they could stably cover large acetabular defects and, at the same time, they matched the implant site. The prosthesis in this study is equivalent to a partial acetabular component; maximizing the preservation of the remaining bone after placement is key. Based on Lagoa et al. [20], they developed a 3D printed repair material for bone defects that were elastic to avoid stress occlusion, had a supporting effect, were biocompatible and could be partially biodescended. Combined with our research, these materials can be used to reconstruct the posterior acetabular wall and maintain bone mass in the posterior acetabular wall, and the properties of partially degradable materials can also be appropriate for patients who will need an artificial hip replacement in the future. Thus, the new concept of posterior acetabular wall reconstruction proposed in this study can be summarized as follows: Break up to Make up.

The prognosis for posterior acetabular fractures is unsatisfactory. Currently, it is common to use autologous iliac bone grafting to rebuild the posterior wall of the acetabulum. This procedure increases the surgical incisions, the amount of intraoperative bleeding and the operation time. Autologous iliac bone grafting sometimes fails to completely dissect and reconstruct the posterior acetabular wall, resulting in joint surface spaces or steps. Uneven joint surfaces can lead to postoperative traumatic arthritis [21], and eventually the patient undergoes artificial hip replacement or other secondary surgery. In a retrospective study by Iselin et al. [22], 10 of 33 patients (30%) with acetabular fracture involving the posterior wall developed symptoms of osteoarthritis, of whom 6 (18%) required total hip replacement or hip arthropexy. The personalized acetabular posterior wall prosthesis, polished articular surface and degradable material properties used in this study have the potential to be another ideal treatment modality.

### Limitations

In this study, calculations were performed only on a hemipelvic model, and analysis of the whole pelvis was not performed. This study assumed that the properties of bone materials were isotropic, ignoring other properties of bone, which will inevitably result in differences from actual values. In addition, the acetabulum of the human body is a complex component, including ligaments, the joint capsule, synovial fluid and surrounding soft tissue structures, which will have a certain impact on the force of the acetabulum. This study consisted of only a numerical simulation, which failed to simulate the friction of the acetabular prosthesis on the femoral head. Thus, the wear caused by long-term contact between the acetabular prosthesis and femoral head needs to be further verified by animal experiments.

## Conclusions

In this study, the use of different methods for the internal fixation of posterior acetabular wall fractures was analysed by finite element analysis. According to the results, a PACP is recommended for simple posterior acetabular fractures. In the case of a comminuted posterior acetabular wall fracture, either an NPAWP or BPAWP can be used, depending on the patient’s condition. The TPAWP in this study is not recommended due to the areas of high stress. Regarding the method of acetabular prosthesis design, we propose the concept of “Break up to Make up” as a guide.

## Data Availability

The datasets used and/or analysed during the current study are available from the corresponding author on reasonable request.

## Abbreviations

CT: Computed tomography
CRP: Common reconstruction plate
PACP: Posterior acetabular column plate
NPAWP: Nonflanked posterior acetabular wall prosthesis
BPAWP: Biflanked posterior acetabular wall prosthesis
TPAWP: Triflanked posterior acetabular wall prosthesis
